# Encoding wild fragrance: The role of allelic variants in floral odor emissions

**DOI:** 10.1093/plphys/kiad244

**Published:** 2023-04-21

**Authors:** Lara Pereira, Henning Kirst

**Affiliations:** Plant Physiology, American Society of Plant Biologists, USA; Ecology and Evolutionary Biology, School of Biosciences, University of Sheffield, Sheffield S10 2TN, UK; Plant Physiology, American Society of Plant Biologists, USA; Departamento de Genética, Campus de Excelencia Internacional Agroalimentario ceiA3, Universidad de Córdoba, Córdoba 14071, Spain; Instituto Maimónides de Investigación Biomédica de Córdoba (IMIBIC), Córdoba 14004, Spain

Plants have evolved to synthesize specialized (or secondary) metabolites. In contrast to primary metabolites such as nucleic acids and amino acids, which are directly connected with growth, these specialized metabolites help the plant to interact and survive within their environment (Erb and Kliebenstein 2020). They cover a wide range of functions from antimicrobial, plant-plant interacting, herbivore-repelling, or pollinator-attracting purposes (Erb and Kliebenstein 2020). One of the most diverse and abundant groups of plant metabolites are terpenoids, which participate in numerous processes such as photosynthesis and photoprotection, hormone biosynthesis, and defensive roles (Srividya et al. 2015). Terpene synthases (TPSs), encoded by a gene family of variable size in different plant genomes, are essential in generating the vast structural diversity of terpenoid natural compounds. These enzymes, classified in 7 major clades, went through duplications and divergence events that contributed to their ability to use a range of substrates and generate a diverse set of products (Chen et al. 2011).

Volatile terpenes are often produced in flowers and are major contributors to floral scent (Muhlemann et al. 2014). Floral fragrance has important ecological roles, from pollinator attraction to florivore repellence (Mostafa et al. 2022). Furthermore, scent is a fundamental trait for ornamental flowers, with a huge impact in consumer's satisfaction. Detailed analysis of the TPS variants in ornamental plants can reveal the underlying mechanisms that determine volatile amounts and composition and ultimately allow breeders to modify flower aroma.

**Figure 1:**
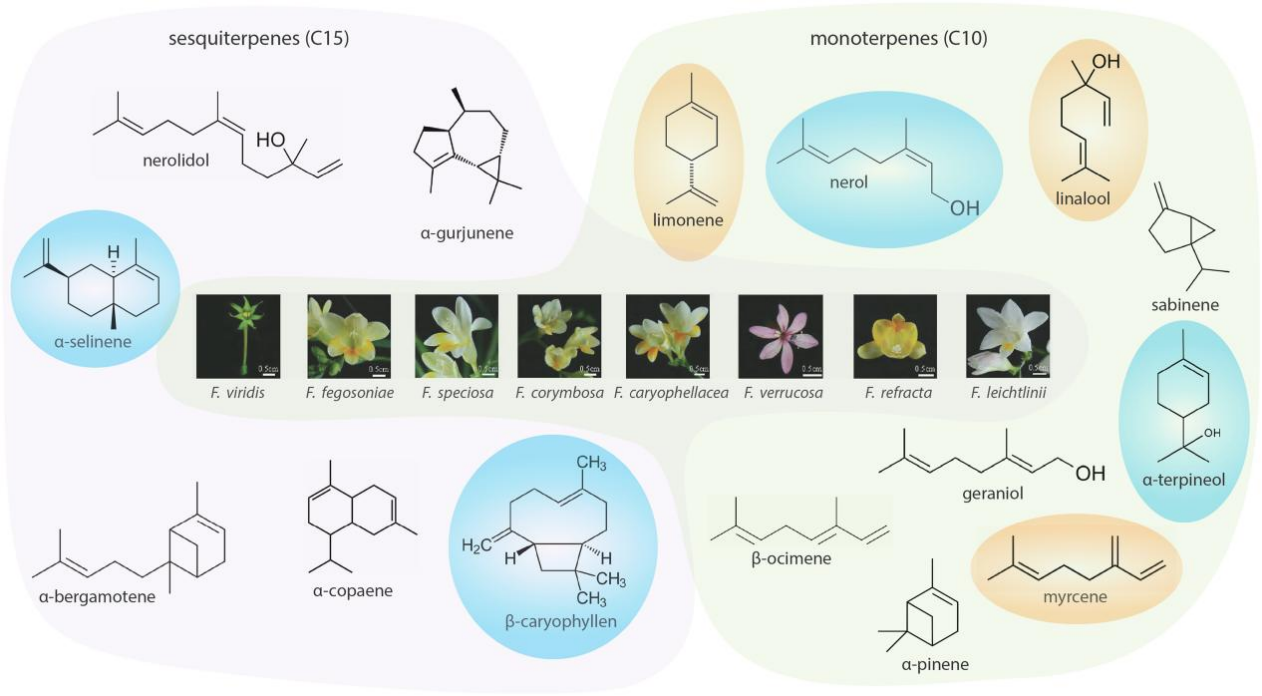
Flowers of wild *Freesia* species and a subset of their diverse floral terpenoids. Terpenoids generated by TPSs from the C15 substrates (E, E) and (Z, Z)-farnesyl diphosphate yield the sesquiterpene (C15) products. Monoterpenes (C10) are generated by TPSs from the C10 geranyl diphosphate (GPP) and neryl diphosphate substrates. The most common and abundant terpenes: limonene, linalool, and myrcene. The terpenes more characteristic in wild *Freesia* species are: β-caryophyllene, α-terpineol, nerol, and α-selinene. Modified from Bao et al. (2023).

In a recent issue of *Plant Physiology*, Bao et al. (2023) used a comprehensive approach combining transcriptomics, metabolomics, and molecular biology to characterize the TPSs responsible for the floral scent in wild Freesia species, including 8 already described and 7 that are novel. The *Freesia* genus, from the Iridaceae family, is widely cultivated as an ornamental cut flower, mainly due to its attractive fragrance. Cultivated varieties belong to *Freesia x hybrida*, likely a hybrid between *Freesia corymbosa* and *Freesia leichtlinii* (Gao *et al*. 2018). The aroma of these cultivated varieties is characterized by high levels of linalool and small quantities of other terpenoids that vary across varieties (Figure 1) (Gao et al. 2018; Weng et al. 2021). However, broader chemo-diversity was found in wild *Freesia* species (Wongchaochant et al. 2005), and characterizing its genetic basis could offer new routes to modify volatile profiles and achieve the scent demanded by the market.

Bao et al. (2023) analyzed whole transcriptome and volatile profiles of the flowers of 8 wild *Freesia* species. Although the flowers of all analyzed *Freesia* species emitted limonene and linalool, the amount and composition of other emitted terpenes differed widely, accounting for 49 different metabolites (Figure 1). The authors identified 15 TPSs, clustered in 3 phylogenetic subgroups. The expression level of these genes, determined by reverse transcription quantitative PCR, showed some correlation with the volatile terpene profiles but could not alone explain the drastic differences among species in volatile composition.

Thus, the authors explored the consequences of allelic variation in the amino acid sequences of TPSs by performing a deeper biochemical analysis. Heterologous expression of TPSs in *Escherichia coli* and their transient expression in *Nicotiana tabacum* leaves both showed that almost all TPSs can utilize the C10 substrates geranyl diphosphate and neryl diphosphate, and 5 were also able to utilize the C15 substrates (E, E) and (Z, Z)-farnesyl diphosphate. Different substrates led to different sets of products, ranging from 1 to up to 14 different terpenoids (Figure 1).

Interestingly, none of the investigated wild *Freesia* species had completely lost the flower scent. In other plant genera, such as *Capsella* and *Petunia*, some wild species have acquired loss-of-function mutations in genes from the benzenoid biosynthetic pathway, resulting in odorless flowers (Raguso 2016). These evolutionary changes are usually associated with a shift in the mating system—for example, from insect-pollinated to self-pollinated flowers. It has been hypothesized that the expansion and functional diversification of TPSs prevented the loss of scent in species in which the floral fragrance is mainly driven by terpenoid volatiles.

Allelic variation from wild *Freesia* species in 3 TPSs affected their enzymatic functionality, altering both the product composition and quantity. The authors used site-directed mutagenesis in combination with metabolic profiling to pinpoint the causative amino acids that impact product specificity of the terpenoid synthases. Notably, changing a single amino acid was sometimes enough to alter volatile emission, which demonstrates the fascinating versatility of TPSs.

This work highlights the incredible diversity, promiscuity, and product plasticity of TPSs that allow plants to succeed in a constantly changing environment. Showing that both gene expression and sequence allelic variation in TPSs contribute to differences in the volatile profiles paves the way to incorporate multiple strategies to breeding pipelines—for example, modulating gene expression to achieve a certain amount of a specific volatile, introgressing wild allelic variants into commercial varieties to incorporate targeted compounds, or even designing synthetic TPSs with the desired properties.
